# Facilitators and Barriers to Digital Self-Management in Older Adults With Depression: COM-B and Theoretical Domain Framework Qualitative Study

**DOI:** 10.2196/79253

**Published:** 2026-04-10

**Authors:** Ziping Zhu, Xinying Lin, Zhi Chen, Hong Li, Rong Lin

**Affiliations:** 1 School of Nursing, Fujian Medical University, Shangjie Campus of Fujian Medical University, Minhou County, Fuzhou, Fujian, 350122, China, 86 15859066807; 2 Fudan Zhongshan Xiamen Hospital, Xiamen, Fujian, China

**Keywords:** geriatric depression, mHealth, Theoretical Domains Framework, TDF, Capability, Opportunity, Motivation, and Behavior model, COM-B model, qualitative descriptive study, mobile health

## Abstract

**Background:**

Depression in older adults presents unique challenges in self-management. Digital tools, such as mobile health (mHealth) apps, have the potential to support this population. This study explored the facilitators and barriers to digital self-management in older adults with depression to inform the design of effective mHealth apps.

**Objective:**

This study aims to explore the facilitators and barriers to digital self-management in older patients with depression with the aim of informing the design and development of mHealth apps for older adults.

**Methods:**

A purposive sampling method was used to recruit 25 older patients with depression from July to September 2024. Semistructured interviews were conducted to capture real-life experiences. Directed content analysis ensured objective and accurate data interpretation, and the Capability, Opportunity, Motivation, and Behavior (COM-B) model and the Theoretical Domains Framework were applied to identify facilitators and barriers related to behavior.

**Results:**

Six themes were identified based on the COM-B model and the Theoretical Domains Framework: perception of illness and accumulation of personal experience; dual challenges of cognitive abilities and physical limitations; integration of digital technologies and acceptance differences; social influences and access to and utilization of support resources; environmental constraints and accommodations; and intertwined influences of beliefs, emotions, and motivation. A total of 13 barriers and 11 facilitators were identified.

**Conclusions:**

Digital self-management in older adults with depression is complex and influenced by multiple interrelated factors. Effective mHealth apps must integrate the cognitive, emotional, and social contexts of patients to provide user-friendly, personalized solutions.

## Introduction

### Background

With the increasing integration of information and communication technologies into health care, mobile health (mHealth) apps have become an important modality within digital health and are increasingly used to support depression management [1-3]. In parallel, self-management has gained prominence in chronic disease care as a means of enabling individuals to actively manage symptoms, improve adherence to treatment, and enhance daily functioning while reducing dependence on formal medical services [4]. Self-management emphasizes personal agency, perceived control, and the translation of therapeutic principles into daily life practices [5,6]. In the context of depression, self-management commonly includes mental health literacy, psychoeducation, medication adherence support, behavioral activation, and cognitive behavioral strategies [7]. Compared with traditional face-to-face services, digital self-management interventions offer advantages, such as improved accessibility, reduced travel burden, scalability, lower costs, and enhanced protection of privacy [8-11]. Evidence from randomized controlled trials suggests that smartphone-based interventions can reduce depressive symptoms [12] and expand access to care, particularly for individuals who face barriers to conventional mental health services [13-16].

The growing interest in digital self-management is particularly relevant given the global burden of depression, one of the most prevalent mental health disorders worldwide and a leading cause of disability. According to the World Health Organization, more than 300 million people globally are affected by depression, and its contribution to disability-adjusted life years continues to increase [17]. Depression is particularly prevalent among older adults and not only directly leads to individual disability but also imposes a substantial economic burden on society [18,19]. Studies project that between the years 2023 and 2032, cumulative all-cause medical expenditure for patients with depression will reach US $309 million, which is 2.6 times more than that for nondepressed individuals [20]. Among individuals aged 60 years and older, global prevalence estimates range from 28.4% to 35.1%, with mortality rates reported to be as high as 9.8% [21,22]. Late-life depression is characterized by high recurrence and mortality and yet remains markedly underdiagnosed and undertreated [23,24]. Beyond its clinical impact, depression imposes considerable economic costs through reduced workforce participation, impaired productivity, increased health care utilization, and the transfer of caregiving responsibilities to family members, placing sustained strain on intergenerational care systems [22,25,26]. Despite this burden, fewer than 10% of individuals with depression worldwide receive any form of treatment, and fewer than 1% receive minimally adequate care [27]. Older adults face additional barriers, including limited service accessibility, transportation difficulties, stigma, and reduced awareness of help-seeking pathways, further increasing the risk of untreated or poorly managed depression [28-31].

Effective self-management is therefore particularly important for older adults with depression [32], although its real-world implementation remains challenging. Depression frequently coexists with multimorbidity, functional decline, and age-related cognitive and sensory changes, increasing the complexity of daily symptom management. Core depressive symptoms, such as low motivation, fatigue, and hopelessness, directly undermine the ability of individuals to initiate and maintain self-management behaviors over time [33]. Although mHealth apps provide tools for symptom monitoring, psychoeducation, and remote support, existing evidence indicates a paradoxical pattern in their use among older adults with late-life depression. Specifically, while mHealth interventions have demonstrated efficacy in controlled trials, such as reducing depressive symptoms and improving sleep quality [34], these benefits have not translated into sustained engagement or effective self-management in the real-world setting, where low uptake, rapid disengagement, and poor long-term adherence are commonly reported [35].

This discrepancy has been attributed to multiple interacting barriers, including fear of mental illness–related stigma, suboptimal user interface designs, and age-related physical and cognitive limitations [36,37]. In the real-world context, additional constraints, such as inadequate digital literacy and privacy concerns, further restrict the adoption and continued use of mHealth tools among older adults [38,39]. In addition, existing apps are rarely tailored to the specific psychosocial needs of this population and often overlook themes central to late-life depression, such as grief, loss, loneliness, and social isolation [39]. However, most studies in this field have relied predominantly on quantitative methods, focusing on the acceptance, usability, or impact on symptom outcomes of technology. Although these approaches are valuable for identifying general influencing factors, such as the digital divide and technophobia, they provide limited insight into how older adults with depression perceive, interpret, and emotionally respond to mHealth interventions in everyday life [40]. In particular, they fail to capture how depressive symptoms dynamically interact with technology use to shape both the initiation and sustained engagement in digital self-management behaviors. Without such experiential and contextual understanding, mHealth interventions risk remaining technically effective in controlled trials but poorly aligned with the needs of users in the real world, perpetuating the persistent gap between effectiveness and participation. Therefore, a qualitative approach is essential to elucidate the mechanisms through which individual, technological, and contextual factors jointly influence engagement in digital self-management among older adults with depression.

### Theoretical Framework

For older adults with depression, digital self-management is not merely a matter of technology adoption but rather a complex process of modifying health behavior. Therefore, understanding this process is crucial for designing effective intervention strategies. Evidence suggests that using theoretical frameworks to identify and analyze determinants of behavior can substantially increase the likelihood of intervention effectiveness [41]. Theoretical frameworks facilitate a comprehensive understanding and interpretation of the complexity of behavior change, providing a solid foundation for effective intervention design [42]. Interventions grounded in behavioral theory models have been shown to be more effective than interventions not based on theory [43]. Therefore, incorporating theoretical frameworks is essential for understanding behavioral changes. Among various theories, the Capability, Opportunity, Motivation, and Behavior (COM-B) model and its associated Theoretical Domains Framework (TDF) have been widely used to identify behavioral facilitators and barriers, providing strong support for designing targeted interventions [44,45]. To comprehensively understand, analyze, and explain the multidimensional factors influencing the adoption and sustained use of digital self-management by older adults, this study adopted the COM-B model and the TDF as its theoretical guiding frameworks.

The COM-B model posits that behavior arises from the interaction of capability, opportunity (social and physical), and motivation (automatic and reflective processes) [46]. Building on this model, the TDF translates these components into 14 operational domains, including knowledge, skills, social influences, and environmental resources, offering a comprehensive and structured tool for systematically identifying and analyzing behavioral determinants [46]. Together, the COM-B model and the TDF form the core of the behavior change wheel, serving as an integrated tool for describing behavior and designing interventions through a concise yet comprehensive analytical structure. Although the COM-B model explains behavior formation through capability, opportunity, and motivation, the TDF further elaborates on these dimensions, enabling a more comprehensive exploration of behavior change possibilities through in-depth interviews and analysis of mHealth self-management among older adults with depression [46]. Compared with other commonly used behavior change theories, the COM-B model and TDF demonstrate advantages in analyzing complex health behaviors. For example, the health belief model emphasizes the perception of disease threat and behavioral benefits by the individual but does not adequately account for social and environmental factors [47]. The theory of planned behavior primarily focuses on individual cognitive factors, such as attitudes and subjective norms, resulting in a relatively narrow perspective [48]. Although the transtheoretical model emphasizes the dynamic nature of behavior change, it lacks systematic identification and classification of specific determinants, limiting its practical applicability [49]. Consequently, the integrated COM-B model and the TDF more comprehensively capture the complex interactions between individual internal factors and external environmental influences on health behaviors, making them particularly suitable for analyzing digital self-management, which is shaped by multiple interacting factors.

In this study, the TDF was used to guide the development of the semistructured interview guide to ensure comprehensive coverage of the theoretical domains that may influence digital self-management behaviors among older adults with depression. Subsequently, qualitative analysis of interview transcripts was conducted to categorize identified barriers and facilitators into relevant TDF domains and ultimately map them onto the three core COM-B components: capability, opportunity, and motivation. This analytical process allowed for a clear and structured presentation of the findings and provided direct theoretical justification and practical guidance to develop feasible and targeted interventions based on the behavior change wheel. Currently, qualitative studies that have applied the combined TDF and COM-B model to explore digital self-management behaviors among older adults with depression in China are scarce. Therefore, this framework provides a solid theoretical foundation for understanding digital self-management behaviors in older adults with depression to inform the design of tailored, context-sensitive digital interventions suitable for Chinese patients.

This study aims (1) to systematically identify the facilitators and barriers that influence the adoption and sustained participation in mHealth interventions among older adults with depression and (2) to map these factors onto the TDF and the COM-B model to elucidate key behavioral determinants, thus informing the development of future strategies to promote the use of mHealth in this population.

## Methods

### Study Design

**Figure 1.**
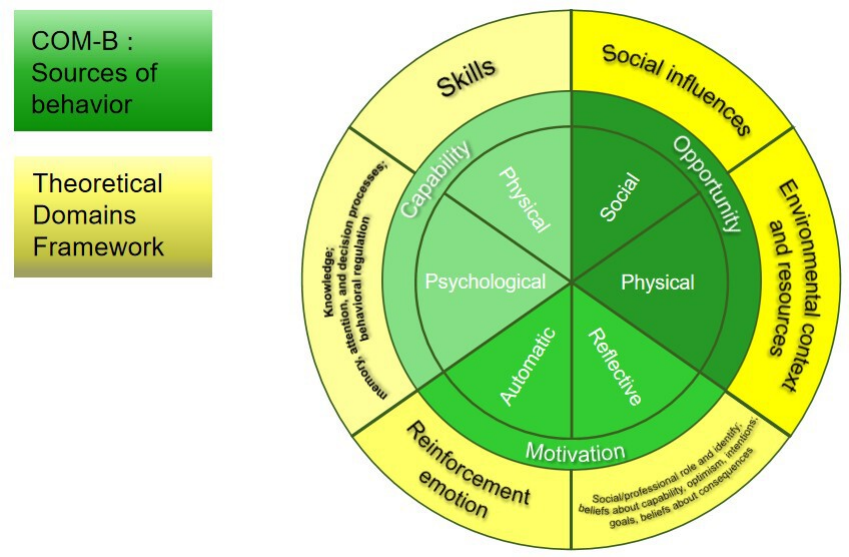
Schematic diagram of the Capability, Opportunity, Motivation, and Behavior (COM-B) model and the Theoretical Domains Framework (TDF).

This study used a descriptive qualitative research methodology that followed the philosophical basis of naturalistic inquiry, which emphasizes the interpretation of patient experience or the presentation of events in simple language [50]. Valuable information can be gathered from understanding an individual’s reactions, thoughts, facilitators, or hindrances to events [51]. To better understand the factors influencing the use of digital tools for self-management in older people with depression, it is important to explore their feelings, experiences, and perceptions related to seeking help. Data were collected through semistructured interviews with open-ended questions. The study used the COM-B model and the TDF to design the outline of the interview and to provide a framework for organizing and analyzing the data (Figure 1). The study was presented in accordance with the COREQ (Consolidated Criteria for Reporting Qualitative Research) checklist (Checklist 1 [52]).

### Study Setting and Recruitment

This study was conducted in 5-star nursing institutions and communities in Fuzhou City, Fujian Province, China. Purposive sampling was used to select interviewees, among those who met the above criteria, and the individuals most likely to provide rich information were selected. The maximum difference sampling strategy was used to account for differences in sex (male or female), age, literacy levels (ranging from primary school to college), economic situation (monthly income starting from <1000 to >5000 CNY [1 CNY=US $0.14]), and disease severity of older patients with depression (mild, moderate, or severe based on 15-item Geriatric Depression Scale scores). Additionally, to ensure diversity in digital experiences, we considered participants’ smartphone ownership and their frequency of use. Based on participants’ self-reported average daily usage duration during the interviews, usage frequency was categorized as low (≤1 hour/day), medium (1‐4 hours/day), or high (≥4 hours/day). The sample size was determined based on the principle of meaning saturation (see Multimedia Appendix 1 for details). After each interview, the research team held regular meetings to review transcripts, identify emerging themes, and discuss whether new information was still being obtained. Saturation was considered achieved when 2 consecutive interviews did not yield new substantive themes related to the research questions, and additional data collection appeared redundant. The final decision on saturation was reached through consensus during these team discussions.

### Inclusion and Exclusion Criteria

The inclusion criteria were as follows: (1) aged 60 years or older, (2) meeting the diagnostic criteria for depression in the *Diagnostic and Statistical Manual of Mental Disorders, Fifth Edition*, (3) a 15-item Geriatric Depression Scale score of 5 or higher, (4) ability to communicate; (5) voluntary participation and provision of informed consent, and (6) no requirement for smartphone ownership or prior experience using smartphones. Including participants with heterogeneous digital skills may increase variability, whereas restricting participation to digitally experienced older adults could limit the transferability of the findings [53,54]. Older adults are not a digitally homogeneous group, and excluding those without smartphone experience may overestimate the feasibility of digital self-management and exacerbate digital inequalities [55]. Including participants with diverse levels of digital engagement allowed for a more realistic assessment of barriers and facilitators in real-world settings. The exclusion criteria were as follows: (1) serious physical illnesses preventing cooperation with the interview, (2) severe mental illnesses preventing participation in the study, and (3) illiterate individuals or those unable to understand basic written information.

### Data Collection

Data were collected between July and September 2024. Information from the literature [56,57] was used for the TDF and was mapped to the components of the COM-B model to develop a semistructured interview outline. Three patients who met the inclusion criteria for preinterviews through the convenience sampling method (preinterview information was not included in the final analysis) were selected, and consultations with relevant experts on issues that arose during the interviews allowed for modifications to the outline. The final outline of the interview was confirmed after rediscussion by the research team and is provided in Multimedia Appendix 2.

Two methods were used for data collection: (1) a demographic survey (covering age, sex, education, and economic situation) and (2) in-depth, semistructured, one-on-one face-to-face interviews. Before the interview, the researcher (ZZ) introduced herself, explained the purpose and significance of the study, outlined the necessity of on-site audio recording and the principle of confidentiality, obtained written informed consent from the interviewees, and assisted the participants in filling out the basic information form. Face-to-face interviews were conducted when the patients were free and in good condition, in a separate room without interference. The interview was conducted with open-ended questions that referred to the outline of the interview but were not restricted to the language or order of the outline. During the interview, attention was paid to recording the patient’s tone of voice, expressions, movements, and other nonverbal behaviors of the patient. The interviewer also listened carefully to the patient’s statements, asked questions and responded to them at the right time, and avoided the use of guiding or suggestive language. The entire interview was recorded, and the key content was noted. The interview lasted 20 to 40 minutes. After the interview, the recorded information was listened to two or three times to ensure accuracy [1], and the information was entered into Microsoft Word.

To enhance the credibility of the data analyses, participants were invited to review and validate the content after the interviews to ensure an accurate understanding and reflection of their views. Detailed records and documentation, including interview outlines, data analysis processes, and rational decision-making, were maintained to facilitate the subsequent review of the data and validate the transparency of the research process.

### Data Analysis

All audio recordings were transcribed into a Word document within 24 hours of the conclusion of the interview. The verbatim transcription of the audio-recorded material was accompanied by the labeling and integration of nonverbal information such as pauses, facial expressions, and body language recorded in the field notes of the interviews. The interview data were anonymized, and the transcripts were numbered with P1, P2, P3,..., Pn codes. Other basic demographic and disease information of the study participants was recorded in a separate document from the transcribed manuscript to ensure systematic and confidential data management.

The transcribed data were analyzed using a directed content analysis approach [58,59], guided by the TDF and the COM-B model. Directed content analysis is an appropriate method when existing theory or previous research can guide initial coding and categorization, while still allowing openness to new insights. The analytical process followed 3 main stages: preparation, organization, and reporting. First, the analysis unit was defined, with meaningful sentences reflecting the experiences, perceived barriers, and facilitators of participants related to mHealth-supported self-management used as the smallest analytical units. The transcripts were then read repeatedly to achieve immersion and gain an overall understanding of the data. In the organization stage, an initial coding framework was developed deductively based on the predefined TDF domains and corresponding COM-B components (capability, opportunity, and motivation). Line-by-line coding was conducted, during which relevant text segments were coded and mapped to the corresponding TDF domains. Similar codes were subsequently grouped into subthemes, which were further organized into higher-order themes aligned with the COM-B model. Although the analytic strategy allowed for inductive coding of data that did not fit the predefined theoretical framework, all the content identified in this study could be meaningfully mapped to the TDF domains and COM-B components. Therefore, the presentation of results reflects primarily findings derived from the directed content analysis. Two co–first authors independently performed the deductive coding and mapping process using established definitions of the COM-B model [46] and the TDF [60]. Any discrepancies were resolved through team discussion to ensure analytical rigor.

### Rigor

The rigor of this study was ensured through systematic steps of directed content analysis and was elaborated upon using the criteria proposed by Colorafi et al [61], specifically across the 4 dimensions of credibility, transferability, confirmability, and consistency [62]. First, to establish credibility, we used in-depth interviews and member checking: all interviews were conducted by the same researcher (ZZ) to maintain consistency in interaction, and rapport was built during interviews to encourage participants to elaborate. Furthermore, we returned key interview summaries to participants for content verification, thereby validating the accuracy of understanding. Second, to ensure transferability, we comprehensively presented the research context, participant characteristics, purposive sampling strategy, and detailed data collection and analysis procedures in the *Methods* section, providing a sufficient basis for other researchers to judge the applicability of this study’s context and findings. Regarding confirmability, we implemented cross-verification among coders: initial coding and the mapping of categories to the COM-B model were independently performed by two researchers (ZZ and XL), followed by review and approval by team members not involved in the initial coding. All analytical decisions and processes were documented for reference, forming a traceable audit trail. Finally, in terms of consistency, the entire study strictly followed the established stages of directed content analysis, and consensus was reached through discussions with the research team at each key analytical junction, thus ensuring the logical coherence and methodological stability of the analytical process.

### Reflexivity

In qualitative research, ensuring the rigor and quality of the study is a core responsibility of the researcher. In this context, researcher reflexivity is regarded as a crucial standard to enhance the credibility of the study [63]. Qualitative researchers use reflexivity to explain how their own subjectivity influences the research process and to make the entire process more transparent [64]. This study was led by 2 experts in the fields of geriatric depression and mental health. All interviews were conducted by ZZ, while the primary coding and mapping analysis to the COM-B model were performed independently by ZZ and XL, with ongoing mutual feedback throughout the process. As the researcher is the primary instrument for data collection and interpretation, throughout the research process, we continuously examined the researcher’s professional background (eg, in clinical psychology and geriatric health) and performance considering preexisting knowledge of digital health technologies, as well as potential assumptions and emotional responses formed during interview interactions, through team discussions, research journals, and periodic reflections. This ongoing reflection aimed to clarify the influence of the researcher’s position on the study. For example, building an empathetic relationship during interviews may enhance the openness of participants to sensitive topics (such as experiences of depression), whereas the researcher’s academic training may also influence the initial interpretation of “self-management” behaviors in the data. By making such reflections explicit and incorporating them into the analytical considerations, we strived to achieve a deeper understanding of the participants’ experiences while enhancing the transparency of the research process and the rigor of interpretation as much as possible.

### Ethical Considerations

This study received approval from the Ethics Committee of Fujian Medical University (number 2024‐87). All participants were clearly informed of the research purpose, their rights, and the principles of data confidentiality. Written or oral informed consent was obtained from each participant prior to the interviews. Audio recordings were securely stored during the research period and were uniformly destroyed upon the completion of the study. Participants had the right to withdraw unconditionally at any stage of the research without any adverse consequences. All collected data were anonymized. Participants who completed all interviews received a gift as a token of appreciation.

## Results

### Study Participants

Twenty-five older adult patients with depression were enrolled in this study, with an age range of 60 to 86 years (mean 76.0, SD 6.1), including 15 female patients and 10 male patients. The education levels ranged from elementary school to undergraduate degree. In terms of severity of depression, 4 cases of severe depression, 12 cases of moderate depression, and 9 cases of mild depression were identified. Regarding smartphone ownership, 22 participants owned a smartphone, whereas 3 did not. Based on the content of the interview, the frequency of smartphone usage was categorized as follows: 14 participants had medium usage frequency, 8 had low usage frequency, and 3 had high usage frequency. This distribution across multiple dimensions supports the representativeness of the sample in capturing a broad spectrum of experiences related to digital self-management among older adults with depression. The basic characteristics of the participants are detailed in Table 1. The average duration of patient interviews in this study was approximately 30 minutes.

**Table 1.** General information on older patients with depression (n=25).

No.	Sex	Age (y)	Educational level	Economic situation (CNY)^a^	Smartphone	Smartphone use frequency	Depression severity
P1	Female	86	College	>5000	Yes	Medium	Moderate
P2	Female	85	Middle or high school	3000‐5000	No	Low	Severe
P3	Female	75	College	>5000	Yes	Medium	Moderate
P4	Male	85	College	>5000	Yes	High	Mild
P5	Female	80	Middle or high school	3000‐5000	Yes	Medium	Mild
P6	Male	80	Middle or high school	3000‐5000	Yes	Medium	Moderate
P7	Female	70	Middle or high school	3000‐5000	Yes	Medium	Mild
P8	Male	75	Primary school	<1000	Yes	Low	Moderate
P9	Female	77	Primary school	<1000	Yes	Medium	Severe
P10	Female	78	Middle or high school	1000‐3000	Yes	Medium	Severe
P11	Female	82	College	>5000	Yes	Medium	Moderate
P12	Female	77	Primary school	>1000	No	Low	Severe
P13	Male	75	Middle or high school	3000‐5000	Yes	Medium	Moderate
P14	Female	73	College	>5000	Yes	High	Mild
P15	Male	78	College	>5000	Yes	Medium	Moderate
P16	Female	82	Middle or high school	3000‐5000	Yes	Low	Mild
P17	Female	78	College	>5000	Yes	Medium	Moderate
P18	Female	82	Middle or high school	>5000	No	Low	Mild
P19	Male	71	Middle or high school	3000‐5000	Yes	High	Mild
P20	Male	60	Middle or high school	3000‐5000	Yes	Low	Mild
P21	Male	74	Middle or high school	1000‐3000	Yes	Low	Moderate
P22	Female	63	College	3000‐5000	Yes	Medium	Moderate
P23	Male	73	College	>5000	Yes	Medium	Moderate
P24	Male	70	Primary school	1000‐3000	Yes	Low	Moderate
P25	Female	72	Primary school	3000‐5000	Yes	Medium	Mild

a An exchange rate of 1 CNY=US $0.14 is applicable.

### Key Themes and Related Areas

#### Overview

**Figure 2.**
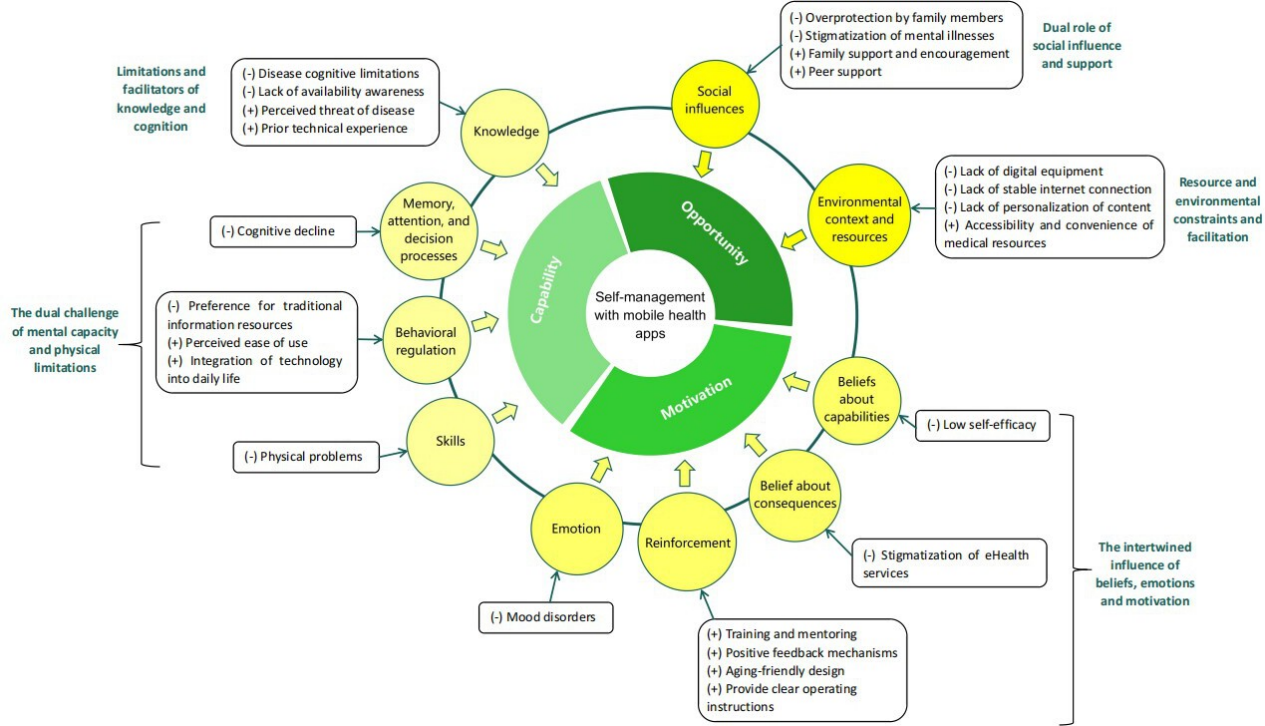
Mapping of themes to the Capability, Opportunity, Motivation, and Behavior (COM-B) model and the Theoretical Domains Framework (TDF).

To improve theoretical clarity, the results are organized according to the COM-B model as the primary analytical hierarchy. Within each COM-B model component (capability, opportunity, motivation), relevant TDF domains and empirically derived themes are presented as subsections. Six themes were identified that were directly related to the 10 TDF domains and 3 COM-B components. The 10 TDF domains were “knowledge”; “memory, attention and decision processes”; “behavioral regulation”; “skills”; “social influences”; “environmental context and resources”; “belief about consequences”; “belief about capabilities”; “reinforcement”; and “emotion,” whereas TDF domains not identified as relevant to the use of mHealth apps to support self-management behaviors were “optimism,” “intentions,” “goals,” and “social or professional roles and identities.” These findings are described in more detail below using the TDF and the corresponding COM-B model (Figure 2). Table 2 provides an overview of the analytical framework, summarizing the facilitators and barriers identified across the TDF domains and COM-B components for participants, whereas detailed theme descriptions and illustrative quotes are presented in Multimedia Appendix 3. The complete data analysis process, from raw interview statements to open codes and subthemes, is provided in Multimedia Appendix 4.

**Table 2.** Facilitators and barriers to digital self-management for older patients with depression, categorized by Theoretical Domains Framework (TDF) domains and Capability, Opportunity, Motivation, and Behavior (COM-B) components.

COM-B components and TDF	Themes	Barriers (number of participants who mentioned them)	Facilitators (number of participants who mentioned them)
Capability			
Psychological capability			
Knowledge	Disease perception and personal experience building	Cognitive limitations of the disease (n=13) Lack of availability awareness (n=8)	Perceived threat of disease (n=7) Prior technical experience (n=13)
Memory, attention, and decision processes	Dual challenges of cognitive function and physical limitations	Cognitive decline (n=16)	Not identified
Behavioral regulation	Digital technology integration and life adaptation	Preference for traditional information resources (n=7) Not identified	Perceived ease of use (n=9) Integration of technology into daily life (n=9)
Physical capability			
Skills	Dual challenges of cognitive function and physical limitations	Restrictions on physical functioning (n=10)	Not identified
Opportunity			
Social opportunity			
Social influences	Access to and utilization of social impact and support resources	Overprotection by family members (n=11) Stigmatization of mental illnesses (n=4)	Family support and encouragement (n=14) Peer support (n=14)
Physical opportunity			
Environmental context and resources	Resource and environmental constraints and facilitation	Lack of digital equipment (n=3) Lack of stable internet connection (n=2) Lack of personalization of content (n=2)	Accessibility and convenience of medical resources (n=10) Not identified Not identified
Motivation			
Reflective motivation			
Belief about capabilities	The intertwined influence of beliefs, emotions, and motivation	Low self-efficacy (n=9)	Not identified
Belief about consequences	Intertwined influence of beliefs, emotions, and motivation	Stigmatization of eHealth services (n=6)	Not identified
Automatic motivation			
Reinforcement	Intertwined influence of beliefs, emotions, and motivation	Not identified	Training and mentoring (n=10) Positive feedback mechanisms (n=8) Aging-friendly design (n=8) Providing clear operating instructions (n=2)
Emotion	Intertwined influence of beliefs, emotions, and motivation	Mood disorders (n=11)	Not identified

#### COM-B: Psychological Capability; TDF: Knowledge (Disease Perception and Personal Experience Building)

Patients’ perceptions about their disease and personal experiences constitute the starting point for their involvement in digital health apps. Due to a lack of accurate understanding of emotional symptoms, some patients (n=13/25, 52%) have a hard time understanding their symptoms and fail to connect their condition with a disease that requires management. This cognitive bias directly influences their motivation to seek help.

*I know I sometimes feel down and bored, but I don't know if this mood is depression, I don't think I can be depressed.*

[P1]

*Most of the time when I am in a bad mood, it is due to my back pain, sometimes my back pain is so bad that my whole body wants to die.*

[P3]

This tendency to attribute depression to physical discomfort or life events diminishes their motivation to actively use self-management tools. Meanwhile, 32% (n=8/25) of the patients demonstrated a lack of awareness or skepticism regarding the value of the app itself, which further dampened their willingness to give it a try.

*I don’t know what these apps can do for me, it feels like just one more thing to worry about.*

[P2]

However, when health threats become tangible, such as a noticeable decrease in physical function, patients’ attitudes often shift. For instance, 28% (n=7/25) of the patients reported that concerns about diminished quality of life could translate into a strong motivation to improve, thereby making them more willing to try new technological approaches.

*I can’t move my feet right now and I’m very limited in what I can do, that’s my biggest struggle and I want to learn some ways to improve my life.*

[P10]

In addition, 52% (n=13/25) of patients noted that prior experience with technology served as a valuable source of confidence, effectively enhancing their acceptance of new technological tools.

*I have no difficulty using these apps, I use a variety of software daily.*

[P4]

*I am quite good at operating my phone, I already have several software programs; more than enough.*

[P19]

Thus, the initial willingness to use such tools is deeply rooted in how individuals perceive their own illness and the technological experience they possess. However, willingness is only the first step. When patients actually begin to use the tools, they immediately face practical challenges shaped by their own capabilities.

#### COM-B: Psychological Capability and Physical Capability; TDF: Memory, Attention, and Decision Processes and Skills (Dual Challenge of Cognitive Abilities and Physical Limitations)

Even with the willingness to use such tools, the cognitive and physical limitations of older patients with depression often create a “mismatch” with the functional design of apps, which constitutes a major obstacle in the adoption process. Approximately 64% (n=16/25) of patients reported that cognitive impairments, such as memory decline and attention deficits, directly challenged the basic abilities required to learn and operate new digital tools.

*I’m a poor reader … and when I use the app, the interface is very easy to accidentally exit, I’ve been taught by volunteers many times before, but I still don’t know how to operate it.*

[P8]

*The brain is not very good, looking at the screen is blurred, memory is bad, easy to forget; the cell phone is used only to make and answer calls. WeChat is not used.*

[P21]

Meanwhile, 40% (n=10/25) of the patients mentioned that the physical symptoms of the disease itself, such as dizziness, visual impairment, and extreme fatigue, made seemingly simple actions like “looking at the screen” and “tapping to operate” unusually strenuous and could even exacerbate their discomfort.

*I’m not well at the moment, I come to the nursing home to get well, and I feel that using these will increase the burden of my illness.*

[P6]

I mainly look at the computer because I think the phone is too small for my eyes and it’s hard to look at it, it’s hard to look at it twice with my eyes.

[P23]

In contrast, several participants (n=14/25, 56%) noted that support from family members or peers helped them continue using digital tools despite physical or cognitive difficulties, as assistance with operation and emotional encouragement reduced frustration during initial attempts.

My eyes are blurry and my fingers aren’t flexible—I almost gave up several times. But my daughter often sits down and guides me through it step by step. In those moments, my heart feels warm and I find the courage to try again.

[P25]

The above challenges that stem from the patients’ own capabilities inevitably affect their perception of the convenience offered by digital tools. Although the process of operation is fraught with frustration, their attitudes toward technology integration also tend to diverge.

#### COM-B: Psychological Capability; TDF: Behavioral Regulation (Digital Technology Integration and Life Adaptation)

Faced with their own ability limitations, patients show a clear divergence in their responses to the integration of digital technology into health management. For instance, 36% (n=9/25) of patients noted that convenient features of apps, such as medication reminders and simple logging, effectively compensate for cognitive deficits, help establish routines, and thereby lead to positive experiences.

*I used to forget to take my medication all the time, but now I have reminders to know when I should take my medication.*

[P9]

*It’s easy and I’d like to have more access to it.*

[P14]

Conversely, for 28% (n=7/25) of patients, these limitations reinforced their reliance on and preference for traditional methods, which they perceived as more reliable and comfortable.

*I think it’s better for older people to read books than to look at their cell phones, there is gold in books.*

[P2]

*I like to read newspapers, I’ve been reading them for more than 40 years. I’m used to it and I seldom use a cell phone.*

[P6]

This variation in acceptance, rooted in individual capabilities, habits, and comfort levels, does not exist in isolation. It is profoundly shaped by the social environment in which patients live, where the attitudes of family members and peers play a crucial role.

#### COM-B: Social Opportunities; TDF: Social Influences (Access to and Utilization of Social Impact and Support Resources)

Patient attitudes toward and attempts to use digital health tools are deeply embedded in their social networks. Positive social influences can serve as a key factor in overcoming self-doubt about their abilities and motivating them to learn. Approximately 56% (n=14/25) of the patients mentioned that encouragement from family members and the sharing of successful experiences by peers provided important external validation and boosted their confidence.

*My wife tried it and said it was good and kept encouraging me to try it too.*

[P6]

*Lao Zhuang shares his experience using the app every day and encourages us to all try it, we all have to learn from him.*

[P5]

However, this social network can also become a constraint. Several participants (n=11/25, 44%) described that family overprotection not only limited their opportunities for hands-on practice but also made them increasingly doubt their own ability to use digital technologies independently, as family members frequently emphasized the difficulty of these tools.

*My kids won’t let me use it, they always tell me to leave it alone, that it’s too much trouble.*

[P8]

*They say the techniques are too complicated and I’m sure I can’t use them, but how do I know if I can use them if I haven’t even tried them yet?*

[P15]

Meanwhile, 16% (n=4/25) of patients mentioned that broader societal prejudices, particularly the stigma surrounding mental illness, could cause them to feel anxious and ashamed even when using health apps in private.

*Depression is an illness that a lot of people don’t understand, especially within nursing homes, they talk about you behind your back all the time.*

[P12]

The power of social support or hindrance ultimately interacts with the material resources and environmental conditions that patients actually possess, jointly determining whether a technology can truly be made “usable.”

#### COM-B: Physical Opportunity; TDF: Environmental Context and Resources (Resource and Environmental Constraints and Facilitation)

Social support can improve willingness; however, if essential material resources and a supportive environment are lacking, actual usage remains unachievable. Approximately 40% (n=10/25) of participants noted that a key advantage of digital health apps lies in their ability to reduce the time and costs associated with traditional health care access.

*I have found that seeing a doctor through my phone and not having to run to the hospital and stand in line is a real time and money saver.*

[P4]

*Now that seeing a doctor and making an appointment with a doctor works on my phone*, *…*

[P14]

However, for some patients, the inability to own a smartphone (n=3/25, 12%) or the lack of a stable internet connection (n=2/25, 8%) still constitutes a fundamental barrier that is difficult to overcome.

*I don’t have a smartphone, and these apps don’t work at all.*

[P2]

*Mainly because of the lack of traffic, my cell phone is used to take and make calls, and I don’t use the other features until I have WiFi at home.*

[P24]

Even after overcoming these hardware barriers, 8% (n=2/25) of patients indicated that if the app content lacked relevance and failed to meet personalized needs, the users’ motivation for continued use diminished rapidly. This reveals a gap between technological offerings and the actual needs of users.

*It’s always the same content and I don’t want to use it anymore.*

[P7]

*There are a lot of features, but I always feel like something is missing and the advice given to me is not tailored to my individual situation, it would be great if it could be adapted to my needs.*

[P15]

The interplay of limited resources and design barriers created major obstacles to sustained use. The experiences of the 3 participants without smartphones underscore that engagement in digital self-management may be constrained not only by individual willingness but also by access to basic devices and connectivity. Rather than expressing resistance, these participants described relying on family members or institutional services for health-related support, suggesting that digital self-management interventions should consider providing foundational access support and proxy-assisted pathways for those lacking personal smartphone access.

#### COM-B: Reflective Motivation, Automatic Motivation; TDF: Beliefs About Capability, Belief About Consequences, Reinforcement, and Emotion (The Intertwined Influence of Beliefs, Emotions, and Motivation)

The entire process of using digital health apps is continuously influenced by a complex interplay of internal beliefs, emotional states, and motivational levels of patients. Approximately 32% (n=8/25) of patients mentioned that positive user experiences, such as receiving immediate feedback and encouragement, significantly enhanced their sense of self-efficacy and willingness to continue usage.

*Even the app is complimenting me on a job well done, which gives me confidence to keep using it.*

[P13]

However, low self-efficacy and the sense of “meaninglessness” inherent in depression can fundamentally erode the internal motivation to engage with such tools.

*I’m so old, I haven’t even figured out how to use an old person’s phone, how can I possibly use a smartphone.*

[P2]

*When I am in a bad mood, I always feel that there is no point in doing anything.*

[P3]

Furthermore, 24% (n=6/25) of patients expressed deep concerns about privacy breaches and online fraud, reflecting a more profound sense of insecurity in the digital age. Such concerns may be amplified by related negative reports in society.

*I don’t dare to use it, there are too many scammers online, in case I lose all my money.*

[P7]

*Worried about information leakage, have to pay attention to the privacy aspect.*

[P22]

Approximately 8% (n=2/25) of patients also clearly expressed that reducing the difficulty of use through aging-friendly design, clear instructions, and continuous training support is crucial for building positive beliefs and motivation.

*Provide training, guide us how to use it a bit, how to deal with the difficulties we encounter with cell phone things. For example, I don’t want this advertisement to appear, but deleting it is troublesome. Sometimes I can’t do it, so I go down and ask young people to click it off.*

[P16]

## Discussion

### Principal Findings

Guided by the COM-B model and the TDF, this qualitative study synthesized how capability, opportunity, and motivation interact to shape the engagement in digital self-management among older adults with depression. Rather than treating barriers and facilitators as isolated determinants, the combined application of the COM-B model and the TDF enabled a mechanistic understanding of why digital self-management engagement is particularly difficult to initiate and sustain in this population. The findings demonstrate that digital self-management engagement extends beyond issues of technology acceptance and is instead embedded within a complex behavioral process influenced by the perception of the disease, cognitive and functional capacity, social context, environmental resources, and depression-related emotional and motivational vulnerability. Although participants generally recognized the potential value of digital tools for mood regulation and daily management, misalignment between individual capability, contextual opportunity, and motivational readiness frequently limited meaningful and sustained engagement.

### Capability: Illness Perception, Cognitive Functioning, and Functional Constraints

At the capability level, digital self-management engagement was shaped less by isolated deficits in technical knowledge and more by how participants understood depression and evaluated their own functional readiness for self-management. Many participants did not conceptualize depressive symptoms as requiring ongoing management, instead attributing emotional distress to aging or physical discomfort, which diminished the perceived relevance of digital self-management tools. This finding refines prior evidence by highlighting that deficits in the TDF “knowledge” domain are not merely informational but also involve inaccurate symptom attribution, which undermines the perceived relevance of digital self-management tools [65-69]. Cognitive decline, fatigue, and attentional limitations further reduced psychological and physical ability, amplifying the effort required to engage with digital tools. However, some participants benefited from simplified functions such as reminders or basic tracking, consistent with evidence on digital prompts facilitating self-management behaviors [70,71]. In contrast, others expressed anxiety and avoidance of digital tools, often rooted in a long-standing reliance on traditional information sources and low confidence in their ability to use technology. This pattern reflects deficits across the TDF domains of “skills” and “beliefs about capabilities” [72-76]. Importantly, our findings suggest that even age-friendly interface principles may be insufficient when depressive symptoms exacerbate cognitive load and reduce tolerance for frustration [39,77-80]. Together, these results indicate that digital self-management capability among older adults with depression should be understood as a dynamic combination of illness perception, cognitive capacity, and functional energy, rather than as a static set of skills.

### Opportunity: Social Influences and Environmental Resources

Social and physical opportunity conditions substantially shaped digital self-management engagement. Consistent with existing literature, supportive involvement from family members and peers enhanced confidence and facilitated behavioral initiation [81-86]. However, this study extends prior findings by demonstrating that social support is not uniformly beneficial. Overprotective family behavior, described by participants as discouraging use or emphasizing technical difficulty, limited hands-on practice and fostered self-doubt regarding their own capabilities, thereby constraining sustained engagement. This nuance refines the TDF “social influences” domain by emphasizing that only autonomy-supportive social environments promote long-term behavioral maintenance [87-89]. With regard to the level of physical opportunity, digital self-management engagement remained constrained by limited access to devices, unstable internet connectivity, insufficient technical support, and financial burden [90-92]. Beyond individual resource limitations, participants’ experiences pointed to broader structural gaps within the digital health ecosystem, which have yet to adequately accommodate the intersecting needs of aging and depression. These findings echo concerns that poorly supported digital interventions may inadvertently widen health inequities [93-95], underscoring the need for system-level adaptations rather than reliance on individual-level solutions.

### Motivation: Beliefs, Emotions, and Reinforcement Mechanisms

Motivation emerged as a central mechanism linking capability and opportunity with digital self-management behavior. Core depressive features, such as apathy, emotional numbness, and reduced reward sensitivity, attenuated intrinsic motivation even when participants cognitively acknowledged potential benefits [96]. Reflective motivation was strongly shaped by beliefs about usefulness, controllability of symptoms, and personal competence, with early negative experiences exerting a disproportionately suppressive effect on continued engagement [97,98]. This finding extends traditional technology acceptance models by highlighting how depressive symptomatology amplifies the motivational consequences of usability failure. Conversely, positive reinforcement, including structured guidance, supportive feedback, and emotionally validating design, was critical in restoring confidence and facilitating habit formation by directly targeting the TDF domains of “reinforcement” and “emotion” [99]. Importantly, motivation did not operate independently: opportunity enhancements such as initial assistance or training partially compensated for limited capability, whereas persistent opportunity barriers nullified motivation regardless of willingness. These interaction patterns help explain why single-component interventions often fail and support the use of the COM-B model as a system-level explanatory model rather than a checklist of determinants [100,101].

Taken together, these findings suggest that older adults with depression may be willing to try digital self-management tools but often face substantial barriers to maintaining engagement over time. Interventions should move beyond simply increasing access to technology and instead address the understanding of the disease, the cognitive burden, and the emotional and motivational vulnerabilities associated with depression. Guided by the COM-B model and TDF, strategies may include targeted psychoeducation to strengthen illness recognition, a simplified and personalized design to reduce cognitive load, structured onboarding and hands-on training to support early success experiences, and supportive feedback mechanisms to maintain confidence during symptom fluctuations. At the system level, embedding digital self-management support within primary care and community-based mental health services, together with basic device and connectivity support, can improve accessibility and reduce avoidable disengagement. Overall, aligning the intervention design with the lived realities of aging and depression can offer a feasible pathway to improve sustained use and long-term self-management outcomes.

### Limitations

As a qualitative study using purposive sampling, this research did not aim to achieve statistical generalizability but rather sought to generate in-depth, contextually grounded insights into the facilitators and barriers shaping digital self-management among older adults with depression. The findings should be considered within the Chinese sociocultural and healthcare context, and while they may not be directly generalizable to all settings, they offer analytical insights that could be transferable to other contexts where aging, depression, and digital health intersect. Second, although this study systematically collected the perspectives and experiences of older patients with depression regarding mHealth management, it did not incorporate professional viewpoints, such as those of health care professionals. These perspectives could have provided valuable insights for the clinical implementation of intervention programs. Third, the interview duration in this study was set at 20 to 40 minutes, considering the cognitive load and attention characteristics of older adults with chronic conditions. Longer interviews could have resulted in fatigue and reduced attention, potentially affecting response quality. Although previous qualitative studies with similar older populations have generally used relatively short interview durations (approximately 15-35 minutes) [102-105], the relatively brief interview time in this study may have somewhat limited the depth and richness of the data, particularly when exploring complex experiences, behavioral factors, and decision-making processes, and may have resulted in some subtle or latent themes not being fully captured.

### Conclusions

This study provides a theory-informed explanation of participation in digital self-management among older adults with depression by applying the COM-B model and the TDF as an integrated analytical lens. The findings highlight that sustained engagement depends on the alignment of capability, opportunity, and motivation rather than on technological availability or willingness. In particular, depression-related cognitive and emotional vulnerability interacts with age-related functional constraints and contextual barriers, helping to explain why engagement is difficult to maintain in real-world settings. These insights emphasize the importance of designing and implementing interventions that are feasible for older adults with depression, including supports that reduce cognitive burden, enable early success, and promote autonomy. At the policy level, strengthening community-based delivery pathways and ensuring easy access to basic digital resources may help prevent the widening of mental health disparities as digital care strategies continue to expand.

## Supplementary material

10.2196/79253 Multimedia Appendix 1 Meaning saturation grid.

10.2196/79253 Multimedia Appendix 2 Interview questions posed to participants based on the Theoretical Domains Framework (TDF) and the Capability, Opportunity, Motivation, and Behavior (COM-B) model.

10.2196/79253 Multimedia Appendix 3 Theme to Capability, Opportunity, Motivation, and Behavior (COM-B) mapping with Theoretical Domains Framework (TDF).

10.2196/79253 Multimedia Appendix 4 From raw interview statements to open codes and subthemes.

10.2196/79253 Checklist 1 COREQ checklist.

## Abbreviations

COM-B: Capability, Opportunity, Motivation, and Behavior
COREQ: Consolidated Criteria for Reporting Qualitative Research
mHealth: mobile health
TDF: Theoretical Domains Framework

## References

[1] Solomon DH, Rudin RS (2020). Digital health technologies: opportunities and challenges in rheumatology. Nat Rev Rheumatol.

[2] Kuwabara A, Su S, Krauss J (2020). Utilizing digital health technologies for patient education in lifestyle medicine. Am J Lifestyle Med.

[3] Ronquillo Y, Meyers A, Korvek SJ (2026). StatPearls [Internet].

[4] Hagerty BM, Bathish MA (2018). Testing the relationship between a self-management intervention for recurrent depression and health outcomes. Appl Nurs Res.

[5] Help and information. Mental Health UK.

[6] Davidson L (2005). Recovery, self management and the expert patient—changing the culture of mental health from a UK perspective. J Ment Health.

[7] Bilsker D, Goldner EM, Anderson E (2012). Supported self-management: a simple, effective way to improve depression care. Can J Psychiatry.

[8] Andersson G, Titov N (2014). Advantages and limitations of Internet-based interventions for common mental disorders. World Psychiatry.

[9] Warmerdam L, Smit F, van Straten A, Riper H, Cuijpers P (2010). Cost-utility and cost-effectiveness of internet-based treatment for adults with depressive symptoms: randomized trial. J Med Internet Res.

[10] Lal S, Adair CE (2014). E-mental health: a rapid review of the literature. Psychiatr Serv.

[11] Richards D, Viganó N (2013). Online counseling: a narrative and critical review of the literature. J Clin Psychol.

[12] Linardon J, Cuijpers P, Carlbring P, Messer M, Fuller-Tyszkiewicz M (2019). The efficacy of app-supported smartphone interventions for mental health problems: a meta-analysis of randomized controlled trials. World Psychiatry.

[13] Mohr DC, Ho J, Duffecy J (2010). Perceived barriers to psychological treatments and their relationship to depression. J Clin Psychol.

[14] Deady M, Johnston D, Milne D (2018). Preliminary effectiveness of a smartphone app to reduce depressive symptoms in the workplace: feasibility and acceptability study. JMIR mHealth uHealth.

[15] Firth J, Torous J, Nicholas J, Carney R, Rosenbaum S, Sarris J (2017). Can smartphone mental health interventions reduce symptoms of anxiety? A meta-analysis of randomized controlled trials. J Affect Disord.

[16] Drissi N, Ouhbi S, Janati Idrissi MA, Ghogho M (2020). An analysis on self-management and treatment-related functionality and characteristics of highly rated anxiety apps. Int J Med Inform.

[17] Depressive disorder (depression). World Health Organization.

[18] Wittchen HU, Jacobi F, Rehm J (2011). The size and burden of mental disorders and other disorders of the brain in Europe 2010. Eur Neuropsychopharmacol.

[19] Sobocki P, Jönsson B, Angst J, Rehnberg C (2006). Cost of depression in Europe. J Ment Health Policy Econ.

[20] Chan VKY, Leung MYM, Chan SSM (2024). Projecting the 10-year costs of care and mortality burden of depression until 2032: a Markov modelling study developed from real-world data. Lancet Reg Health West Pac.

[21] Hu T, Zhao X, Wu M (2022). Prevalence of depression in older adults: a systematic review and meta-analysis. Psychiatry Res.

[22] Cai H, Jin Y, Liu R (2023). Global prevalence of depression in older adults: a systematic review and meta-analysis of epidemiological surveys. Asian J Psychiatr.

[23] Vyas CM, Okereke OI (2020). Late-life depression: a narrative review on risk factors and prevention. Harv Rev Psychiatry.

[24] Park M, Unützer J (2011). Geriatric depression in primary care. Psychiatr Clin North Am.

[25] Greenberg PE, Fournier AA, Sisitsky T, Pike CT, Kessler RC (2015). The economic burden of adults with major depressive disorder in the United States (2005 and 2010). J Clin Psychiatry.

[26] König H, König HH, Konnopka A (2019). The excess costs of depression: a systematic review and meta-analysis. Epidemiol Psychiatr Sci.

[27] Huang Y, Wang Y, Wang H (2019). Prevalence of mental disorders in China: a cross-sectional epidemiological study. Lancet Psychiatry.

[28] van Beljouw IM, Verhaak PF, Cuijpers P, van Marwijk HW, Penninx BW (2010). The course of untreated anxiety and depression, and determinants of poor one-year outcome: a one-year cohort study. BMC Psychiatry.

[29] Donohue JM, Pincus HA (2007). Reducing the societal burden of depression: a review of economic costs, quality of care and effects of treatment. Pharmacoeconomics.

[30] Collins KA, Westra HA, Dozois DJA, Burns DD (2004). Gaps in accessing treatment for anxiety and depression: challenges for the delivery of care. Clin Psychol Rev.

[31] Chekroud AM, Foster D, Zheutlin AB (2018). Predicting barriers to treatment for depression in a U.S. national sample: a cross-sectional, proof-of-concept study. Psychiatr Serv.

[32] Polacsek M, Boardman GH, McCann TV (2021). A theory on the components of depression self-management in older adults. Qual Health Res.

[33] Sherdell L, Waugh CE, Gotlib IH (2012). Anticipatory pleasure predicts motivation for reward in major depression. J Abnorm Psychol.

[34] Chou YH, Lin C, Lee SH, Chang Chien YW, Cheng LC (2023). Potential mobile health applications for improving the mental health of the elderly: a systematic review. Clin Interv Aging.

[35] Gould CE, Loup J, Kuhn E (2020). Technology use and preferences for mental health self-management interventions among older veterans. Int J Geriatr Psychiatry.

[36] Almulhem JA (2023). Factors, barriers, and recommendations related to mobile health acceptance among the elderly in Saudi Arabia: a qualitative study. Healthcare (Basel).

[37] Josephine K, Josefine L, Philipp D, David E, Harald B (2017). Internet- and mobile-based depression interventions for people with diagnosed depression: a systematic review and meta-analysis. J Affect Disord.

[38] Ahmad NA, Mat Ludin AF, Shahar S, Mohd Noah SA, Mohd Tohit N (2020). Willingness, perceived barriers and motivators in adopting mobile applications for health-related interventions among older adults: a scoping review protocol. BMJ Open.

[39] Yin R, Rajappan D, Martinengo L (2025). Depression self-care apps' characteristics and applicability to older adults: systematic assessment. J Med Internet Res.

[40] Posselt J, Lander J, Dierks ML (2024). Health literacy promotion and digital interventions for depressive disorders. Health Lit Res Pract.

[41] Herber OR, Atkins L, Störk S, Wilm S (2018). Enhancing self-care adherence in patients with heart failure: a study protocol for developing a theory-based behaviour change intervention using the COM-B behaviour model (ACHIEVE study). BMJ Open.

[42] Noar SM, Zimmerman RS (2005). Health Behavior Theory and cumulative knowledge regarding health behaviors: are we moving in the right direction?. Health Educ Res.

[43] Buchanan H, Newton JT, Baker SR, Asimakopoulou K (2021). Adopting the COM-B model and TDF framework in oral and dental research: a narrative review. Community Dent Oral Epidemiol.

[44] Michie S, Johnston M, Abraham C (2005). Making psychological theory useful for implementing evidence based practice: a consensus approach. Qual Saf Health Care.

[45] Taylor N, Lawton R, Conner M (2013). Development and initial validation of the determinants of physical activity questionnaire. Int J Behav Nutr Phys Act.

[46] Michie S, van Stralen MM, West R (2011). The behaviour change wheel: a new method for characterising and designing behaviour change interventions. Implement Sci.

[47] Janz NK, Becker MH (1984). The health belief model: a decade later. Health Educ Q.

[48] Armitage CJ, Conner M (2001). Efficacy of the theory of planned behaviour: a meta-analytic review. Br J Soc Psychol.

[49] Brug J, Conner M, Harré N, Kremers S, McKellar S, Whitelaw S (2005). The Transtheoretical Model and stages of change: a critique: observations by five commentators on the paper by Adams, J. and White, M. (2004) why don’t stage-based activity promotion interventions work?. Health Educ Res.

[50] Dehlholm-Lambertsen B, Maunsbach M (1997). Qualitative methods in empirical health research. III. The individual in-depth interview [Article in Danish]. Nord Med.

[51] Sandelowski M (2000). Whatever happened to qualitative description?. Res Nurs Health.

[52] Tong A, Sainsbury P, Craig J (2007). Consolidated criteria for reporting qualitative research (COREQ): a 32-item checklist for interviews and focus groups. Int J Qual Health Care.

[53] Chang SJ, Yang E, Ryu H, Kim HJ, Yoon JY (2018). Cross-cultural adaptation and validation of the eHealth literacy scale in Korea. Korean J Adult Nurs.

[54] Clohessy S, Kempton C, Ryan K, Grinbergs P, Elliott MT (2024). Exploring older adults’ perceptions of using digital health platforms for self-managing musculoskeletal health conditions: focus group study. JMIR Aging.

[55] Money A, Hall A, Harris D, Eost-Telling C, McDermott J, Todd C (2024). Barriers to and facilitators of older people’s engagement with web-based services: qualitative study of adults aged >75 years. JMIR Aging.

[56] Flannery C, McHugh S, Anaba AE (2018). Enablers and barriers to physical activity in overweight and obese pregnant women: an analysis informed by the theoretical domains framework and COM-B model. BMC Pregnancy Childbirth.

[57] Timlin D, McCormack JM, Simpson EE (2021). Using the COM-B model to identify barriers and facilitators towards adoption of a diet associated with cognitive function (MIND diet). Public Health Nutr.

[58] Elo S, Kyngäs H (2008). The qualitative content analysis process. J Adv Nurs.

[59] Hsieh HF, Shannon SE (2005). Three approaches to qualitative content analysis. Qual Health Res.

[60] Cane J, O’Connor D, Michie S (2012). Validation of the theoretical domains framework for use in behaviour change and implementation research. Implement Sci.

[61] Colorafi KJ, Evans B (2016). Qualitative descriptive methods in health science research. HERD.

[62] Sullivan-Bolyai S, Bova C (2021). Qualitative Description: a “How-To” guide. https://repository.escholarship.umassmed.edu/server/api/core/bitstreams/75fe4c77-3447-4214-9286-ead665fc97e6/content.

[63] Olmos-Vega FM, Stalmeijer RE, Varpio L, Kahlke R (2022). A practical guide to reflexivity in qualitative research: AMEE Guide No. 149. Med Teach.

[64] Dodgson JE (2019). Reflexivity in qualitative research. J Hum Lact.

[65] Kim B, Jeong CH, Blood E (2025). Multilevel factors influencing eHealth adoption among older adults during the pandemic. Front Public Health.

[66] Pocklington C (2017). Depression in older adults. Br J Med Pract.

[67] Frost R, Nair P, Aw S (2020). Supporting frail older people with depression and anxiety: a qualitative study. Aging Ment Health.

[68] Rong J, Cheng P, Li D, Wang X, Zhao D (2024). Global, regional, and national temporal trends in prevalence for depressive disorders in older adults, 1990-2019: an age-period-cohort analysis based on the global burden of disease study 2019. Ageing Res Rev.

[69] Mosleh SM, Almalik MM (2016). Illness perception and adherence to healthy behaviour in Jordanian coronary heart disease patients. Eur J Cardiovasc Nurs.

[70] Orr JA, King RJ (2015). Mobile phone SMS messages can enhance healthy behaviour: a meta-analysis of randomised controlled trials. Health Psychol Rev.

[71] Arensman R, Kloek C, Pisters M, Koppenaal T, Ostelo R, Veenhof C (2022). Patient perspectives on using a smartphone app to support home-based exercise during physical therapy treatment: qualitative study. JMIR Hum Factors.

[72] Wu Y, Wen J, Wang X (2022). Associations between e-health literacy and chronic disease self-management in older Chinese patients with chronic non-communicable diseases: a mediation analysis. BMC Public Health.

[73] Kim KA, Kim YJ, Choi M (2018). Association of electronic health literacy with health-promoting behaviors in patients with type 2 diabetes: a cross-sectional study. Comput Inform Nurs.

[74] Zhou M, Zhao L, Kong N, Campy KS, Qu S, Wang S (2019). Factors influencing behavior intentions to telehealth by Chinese elderly: an extended TAM model. Int J Med Inform.

[75] Lin CY, Ganji M, Griffiths MD, Bravell ME, Broström A, Pakpour AH (2020). Mediated effects of insomnia, psychological distress and medication adherence in the association of eHealth literacy and cardiac events among Iranian older patients with heart failure: a longitudinal study. Eur J Cardiovasc Nurs.

[76] Castarlenas E, Sánchez-Rodríguez E, Roy R (2021). Electronic health literacy in individuals with chronic pain and its association with psychological function. Int J Environ Res Public Health.

[77] Cullen R (2001). Addressing the digital divide. Online Inf Rev.

[78] Wilson J, Heinsch M, Betts D, Booth D, Kay-Lambkin F (2021). Barriers and facilitators to the use of e-health by older adults: a scoping review. BMC Public Health.

[79] Chatsatrian M, Kunde K, Bosompem J (2025). Usability evaluation of digital health applications for older people with depressive disorders: prospective observational study in a mixed methods design. JMIR Hum Factors.

[80] Guidance on applying WCAG 2 to non-web information and communications technologies (WCAG2ICT). World Wide Web Consortium (W3C).

[81] Quirke E, Klymchuk V, Suvalo O, Bakolis I, Thornicroft G (2021). Mental health stigma in Ukraine: cross-sectional survey. Glob Ment Health (Camb).

[82] Yu H, Gao Y, Tong T (2022). Self-management behavior, associated factors and its relationship with social support and health literacy in patients with obstructive sleep apnea-hypopnea syndrome. BMC Pulm Med.

[83] Zhang XN, Qiu C, Zheng YZ, Zang XY, Zhao Y (2020). Self-management among elderly patients with hypertension and its association with individual and social environmental factors in China. J Cardiovasc Nurs.

[84] Tang R, Luo D, Li B, Wang J, Li M (2023). The role of family support in diabetes self-management among rural adult patients. J Clin Nurs.

[85] Jo A, Ji Seo E, Son YJ (2020). The roles of health literacy and social support in improving adherence to self-care behaviours among older adults with heart failure. Nurs Open.

[86] Costa ALS, Heitkemper MM, Alencar GP, Damiani LP, Silva RMD, Jarrett ME (2017). Social support is a predictor of lower stress and higher quality of life and resilience in Brazilian patients with colorectal cancer. Cancer Nurs.

[87] Tovar E, Rayens MK, Gokun Y, Clark M (2015). Mediators of adherence among adults with comorbid diabetes and depression: the role of self-efficacy and social support. J Health Psychol.

[88] Lorig KR, Ritter P, Stewart AL (2001). Chronic disease self-management program: 2-year health status and health care utilization outcomes. Med Care.

[89] Volz M, Möbus J, Letsch C, Werheid K (2016). The influence of early depressive symptoms, social support and decreasing self-efficacy on depression 6 months post-stroke. J Affect Disord.

[90] Shi Z, Du X, Li J, Hou R, Sun J, Marohabutr T (2024). Factors influencing digital health literacy among older adults: a scoping review. Front Public Health.

[91] Xie B, Charness N, Fingerman K, Kaye J, Kim MT, Khurshid A (2020). When going digital becomes a necessity: ensuring older adults’ needs for information, services, and social inclusion during COVID-19. J Aging Soc Policy.

[92] Anderson M, Perrin A (2017). Tech adoption climbs among older adults. https://www.pewresearch.org/internet/wp-content/uploads/sites/9/2017/05/PI_2017.05.17_Older-Americans-Tech_FINAL.pdf.

[93] Cornejo Müller A, Wachtler B, Lampert T (2020). Digital Divide – Soziale Unterschiede in der Nutzung digitaler Gesundheitsangebote [Article in German]. Bundesgesundheitsbl.

[94] Hale TM, Chou WY, Cotten SR (2018). eHealth Current Evidence, Promises, Perils and Future Directions.

[95] Weiss D, Rydland HT, Øversveen E, Jensen MR, Solhaug S, Krokstad S (2018). Innovative technologies and social inequalities in health: a scoping review of the literature. PLoS One.

[96] Ma R, Wang Y, Wang XQ, Yu K, Zhang CC, Zhou YQ (2023). Analysis of hindering and facilitating factors of help-seeking behavior in schizophrenia based on COM-B model: a descriptive qualitative study. BMC Psychiatry.

[97] Tsai JM, Cheng MJ, Tsai HH, Hung SW, Chen YL (2019). Acceptance and resistance of telehealth: the perspective of dual-factor concepts in technology adoption. Int J Inf Manage.

[98] Knapova L, Klocek A, Elavsky S (2020). The role of psychological factors in older adults’ readiness to use eHealth technology: cross-sectional questionnaire study. J Med Internet Res.

[99] Taylor SE, Stanton AL (2007). Coping resources, coping processes, and mental health. Annu Rev Clin Psychol.

[100] Xia J, Merinder LB, Belgamwar MR (2011). Psychoeducation for schizophrenia. Cochrane Database Syst Rev.

[101] Lee WH, Lee WK (2017). Cognitive rehabilitation for patients with schizophrenia in Korea. Asian J Psychiatr.

[102] Qiu YF, Liu JL, Zeng LJ (2025). Older adults’ perceptions and needs regarding mental health WeChat applet: a qualitative study. BMC Psychiatry.

[103] Chen S, Yang K, Ko A, Giovannucci E, Stults-Kolehmainen M, Yang L (2025). Facilitators and barriers of reducing sedentary behavior in sedentary and non-sedentary older adults: a descriptive qualitative study based on the COM-B model and TDF. BMC Public Health.

[104] Shen A, Wu P, Qiang W (2025). Breast cancer survivors’ experiences of barriers and facilitators to lymphedema self-management behaviors: a theory-based qualitative study. J Cancer Surviv.

[105] Schöne C, Fuchs TI, Kiselev J (2025). Facilitators and barriers to participation in prehabilitation prior to orthopaedic elective surgery—a qualitative study with elderly (pre-)frail patients. BMC Geriatr.

