# Supplementary material for: Facilitators and Barriers to Digital Self-Management in Older Adults With Depression: COM-B and Theoretical Domain Framework Qualitative Study
Source: JMIR Aging. 2026 Apr 10;9:e79253. doi: 10.2196/79253 (PMC13068307; doi:10.2196/79253)
Supplement: Multimedia Appendix 1 [file aging-v9-e79253-s001.docx]

****Multimedia Appendix 1****

****Meaning Saturation Grid****

| ****Meaning Unit**** | ****Meaning of Meaning Unit**** | | | |
| --- | --- | --- | --- | --- |
|  | ****Interviews 1-10**** | ****Interviews 11-20**** | ****Interviews 21-25**** | ****After 25 Interviews**** |
| **Disease Perception and Personal Experience Building** | **Disease cognitive limitations (1)** |  |  |  |
|  | **Lack of availability awareness (2)** |  |  |  |
|  | **Perceived threat of disease (10)** |  |  |  |
|  | **Prior technical experience (4)** |  |  |  |
| Dual Challenges of Cognitive Function and Physical Limitations | Cognitive decline  **(8)** |  |  |  |
|  | Restrictions on physical functioning **(6)** |  |  |  |
| Digital Technology Integration and Life Adaptation | Preference for traditional information resources**(2)** | Perceived ease of use **(14)** |  |  |
|  | Integration of technology into daily life **(9)** |  |  |  |
| Access to and Utilization of Social Impact and Support Resources | Overprotection by family members**(8)**  Peer support **(5)** | Stigmatization of mental illnesses**(12)** |  |  |
|  | Family support and encouragement **(6)** |  |  |  |
| Resource and Environmental Constraints and Facilitation | Lack of digital equipment **(2)** | Lack of personalization of content **(15)** | Lack of stable internet connection (24) |  |
|  | Accessibility and convenience of medical resources **(4)** |  |  |  |
| The Intertwined Influence of Beliefs, Emotions and Motivation | Low self-efficacy **(2)** | Training and mentoring **(16)** |  |  |
|  | Stigmatization of eHealth services **(7)** | Positive feedback mechanisms **(13)** |  |  |
|  | Mood disorders **(3)** | Aging-friendly design **(16)** |  |  |
|  |  | Provide clear operating instructions **(16)** |  |  |
