# Supplementary material for: Facilitators and Barriers to Digital Self-Management in Older Adults With Depression: COM-B and Theoretical Domain Framework Qualitative Study
Source: JMIR Aging. 2026 Apr 10;9:e79253. doi: 10.2196/79253 (PMC13068307; doi:10.2196/79253)
Supplement: Multimedia Appendix 2 [file aging-v9-e79253-s002.docx]

**Multimedia Appendix 2**

**Interview questions posed to participants based on the TDF and the COM-B model**

| **COM-B**  **Model** | **TDF**  **Framework** | **Outline of An Interview** |
| --- | --- | --- |
| Psychological capacity | Knowledge | ·Can you talk about your knowledge of depression?  ·How do you feel about using mobile health apps to manage your health? |
|  | Memory, attention and desicion processes | ·Do you feel that memory or attention deficit affects the use of mobile health apps? |
|  | Behavioural regulation | ·What factors do you think facilitate or hinder your ability to use mHealth apps? |
| Physical  capability | Skills | ·Do you believe that physical health conditions affect your ability to use mHealth apps? |
| Social opportunities | Social influences | ·Do the people around you (including family, friends, healthcare professionals, etc.) influence your use of mobile health apps? |
| Physical opportunity | Environmental context and resources | ·What are some of the factors in your surroundings that affect your use of mHealth apps? |
| Reflective motivation | Beliefs about capabilities | ·How confident are you that you have mastered the use of mobile health apps? |
|  | Belief about consequences | ·How do you think mobile health apps will affect your health? |
|  | Social/professional roles and identities | ·Does the role you play in society as an older adult affect your attitudes toward the acceptance and use of mHealth apps? |
|  | Optimism | ·What is your mindset when it comes to using mobile health apps? |
|  | Goals/ Intentions | ·What specific intentions or goals do you hope to accomplish by using the mHealth app? |
| Automatic motivation | Reinforcement | What incentives/disincentives do you think would motivate you to use mHealth apps better? |
|  | Emotion | How do you think emotions affect your use of mobile health apps? |
