# Supplementary material for: Facilitators and Barriers to Digital Self-Management in Older Adults With Depression: COM-B and Theoretical Domain Framework Qualitative Study
Source: JMIR Aging. 2026 Apr 10;9:e79253. doi: 10.2196/79253 (PMC13068307; doi:10.2196/79253)
Supplement: Multimedia Appendix 3 [file aging-v9-e79253-s003.docx]

**Multimedia Appendix 3**

**Theme to COM-B mapping with TDF**

| **COM-B components** | | **TDF Frameworks** | **Themes** | **Sub-themes** | **Representative Quotes** |
| --- | --- | --- | --- | --- | --- |
| **Capability** | **Psychological capacity** | Knowledge | Disease Perception and Personal Experience Building | (－) Disease cognitive limitations | *“I know I feel down and bored sometimes, but I don't know if this mood is depression, I don't think I can be depressed.” (P1)* |
|  |  |  |  | (－) Lack of availability awareness | *“I don't know what these apps can do for me, it feels like just one more thing to worry about.” (P2)* |
|  |  |  |  | (＋) Perceived threat of disease | *“I can't move my feet right now and I'm very limited in what I can do, that's my biggest struggle and I want to learn some ways to improve my life.” (P10)* |
|  |  |  |  | (＋) Prior technical experience | *“I am quite good at operating my phone, I already have several software, more than enough.” (P19)* |
|  |  | Memory, attention and desicion progresses | Dual Challenges of Cognitive Function and Physical Limitations | (－) Cognitive decline | *“I'm a poor reader, … and when I use the app the interface, it is very easy to accidentally exit, I've been taught by volunteers many times before, but I still don't know how to operate it.” (P8)* |
|  |  | Behavioural regulation | **Digital Technology Integration and Life Adaptation** | (－) Preference for traditional information resources | *“I think it's better for older people to read books than to look at their cell phones, there is gold in books.” (P2)* |
|  |  |  |  | (＋) Perceived ease of use | *“It's easy and I'd like to have more access to it.” (P14)* |

**Theme to COM-B mapping with TDF frameworks (continue)**

| **COM-B components** | | **TDF Frameworks** | **Themes** | **Sub-themes** | **Representative Quotes** |
| --- | --- | --- | --- | --- | --- |
|  |  |  |  | (＋) Integration of technology into daily life | *“I used to forget to take my medication all the time, but now I have reminders to know when I should take my medication.” (P9)* |
|  | **Physical capability** | Skills | Dual Challenges of Cognitive Function and Physical Limitations | (－) Restrictions on physical functioning | *“I mainly look at the computer because I think the phone is too small for my eyes and it's hard to look at it, it's hard to look at it twice with my eyes.” (P23)*. |
| **Opportunity** | **Social opportunity** | Social influences | Access to and Utilization of Social Impact and Support Resources | (－) Overprotection by family members | *“My kids won't let me use it, they always tell me to leave it alone, that it's too much trouble.” (P8)* |
|  |  |  |  | (－) Stigmatization of mental illnesses | *“Depression is an illness that a lot of people don't understand, especially within nursing homes, they talk about you behind your back all the time.” (P12)* |
|  |  |  |  | (＋) Family support and encouragement | *“My wife tried it and said it was good and kept encouraging me to try it too.” (P6)* |
|  |  |  |  | (＋) Peer support | *“Lao Zhuang shares his experience of using the app every day and encourages us to let us all try it, we all have to learn from him.” (P5)* |
|  | **Physical opportunity** | Environmental context and resources | Resource and Environmental Constraints and Facilitation | (－) Lack of digital equipment | *“I don't have a smartphone, and these apps don't work at all.” (P2)* |

**Theme to COM-B mapping with TDF frameworks (continue)**

| **COM-B components** | | **TDF Frameworks** | **Themes** | **Sub-themes** | **Representative Quotes** |
| --- | --- | --- | --- | --- | --- |
|  |  |  |  | (－) Lack of stable internet connection | *“Mainly because of the lack of traffic, my cell phone is used take and make calls, and I don't use the other features until I have WIFI at home.” (P24)* |
|  |  |  |  | (－) Lack of personalization of content | *“There are a lot of features, but I always feel like something is missing and the advice given to me is not tailored to my individual situation, it would be great if it could be adapted to my needs.” (P15)* |
|  |  |  |  | (＋) Accessibility and convenience of medical resources | *“I have found that seeing a doctor through my phone and not having to run to the hospital and stand in line is a real time and money saver.” (P4)* |
| **Motivation** | **Reflective motivation** | Beliefs about capability | The Intertwined Influence of Beliefs, Emotions and Motivation | (－) Low self-efficacy | *“I'm so old, I haven't even figured out how to use an old person's phone, how can I possibly use a smartphone.” (P2)* |
|  |  | Belief about consequences | The Intertwined Influence of Beliefs, Emotions and Motivation | (－) Stigmatization of eHealth services | *“I don't dare to use it, there are too many scammers online, in case I get all my money.” (P7)* |
|  | **Automatic motivation** | Reinforcement | The Intertwined Influence of Beliefs, Emotions and Motivation | (＋) Training and mentoring | *“Provide training, guide us how to use it a bit, how to deal with the difficulties we encounter with cell phone things. For example, I don’t want this advertisement to appear, to delete it, messy, sometimes I can't do it, so I go down and ask the young people to click it off.” (P16)* |

**Theme to COM-B mapping with TDF frameworks (continue)**

| **COM-B components** | | **TDF Frameworks** | **Themes** | **Sub-themes** | **Representative Quotes** |
| --- | --- | --- | --- | --- | --- |
|  |  |  |  | (＋) Positive feedback mechanisms | *“Even the app is complimenting me on a job well done, which gives me confidence to keep using it.” (P13)* |
|  |  |  |  | (＋) Aging-friendly design | *“Provide training, guide us how to use it a bit, how to deal with the difficulties we encounter with cell phone things. For example, I don’t want this advertisement to appear, to delete it, messy, sometimes I can't do it, so I go down and ask the young people to click it off.” (P16)* |
|  |  |  |  | (＋) Provide clear operating instructions | *“Provide training, guide us how to use it a bit, how to deal with the difficulties we encounter with cell phone things. For example, I don’t want this advertisement to appear, to delete it, messy, sometimes I can't do it, so I go down and ask the young people to click it off.” (P16)* |
|  |  | Emotion | The Intertwined Influence of Beliefs, Emotions and Motivation | (－) Mood disorders | *“When I am in a bad mood, I always feel that there is no point in doing anything.” (P3)* |
