# Supplementary material for: Facilitators and Barriers to Digital Self-Management in Older Adults With Depression: COM-B and Theoretical Domain Framework Qualitative Study
Source: JMIR Aging. 2026 Apr 10;9:e79253. doi: 10.2196/79253 (PMC13068307; doi:10.2196/79253)
Supplement: Multimedia Appendix 4 [file aging-v9-e79253-s004.docx]

### ****Multimedia Appendix 4****

### ****From Raw Interview Statements to Open Codes and Sub-themes****

To ensure methodological rigor and a clear audit trail, this section presents the full analytical pathway from raw participant statements to final theoretical constructs. The table below details the ****open coding**** process, lists all ****72 initial codes**** derived directly from the data, and maps each code definitively to the corresponding ****sub-theme**** within the established COM-B/TDF framework.

| **No.** | **Raw Participant Statement (Representative)** | **Initial Open Code** | **Mapped Sub-theme** | **Themes** | **COM-B components** |
| --- | --- | --- | --- | --- | --- |
| 1 | P1: “I know I feel down and bored sometimes, but I don't know if this mood is depression, I don't think I can be depressed.” | 1. Ambiguous recognition of depressive symptoms | (－) Disease cognitive limitations | Disease Perception and Personal Experience Building | Capacity |
|  |  | 2. Self-denial of having the illness | (－) Disease cognitive limitations | Disease Perception and Personal Experience Building | Capacity |
| 2 | P2: “I don't know what these apps can do for me, it feels like just one more thing to worry about.” | 3. Unawareness of mHealth app value | (－) Lack of availability awareness | Disease Perception and Personal Experience Building | Capacity |
|  |  | 4. Perceived as an additional burden | (－) Lack of availability awareness | Disease Perception and Personal Experience Building | Capacity |
| 3 | P12: “When I have a health problem, I feel like I have to go to the hospital to see a doctor, I don't believe that apps have a role in treating diseases.” | 5. Trust only in traditional healthcare pathways | (－) Lack of availability awareness | Disease Perception and Personal Experience Building | Capacity |
|  |  | 6. Skepticism about digital intervention effectiveness | (－) Lack of availability awareness | Disease Perception and Personal Experience Building | Capacity |
| 4 | P3: “Most times when I am in a bad mood it is because of my back pain, sometimes my back pain is so bad that my whole body wants to die.” | 7. Attribution of mood issues to somatic pain | (－) Disease cognitive limitations | Disease Perception and Personal Experience Building | Capacity |
| 5 | P10: “I can't move my feet right now and I'm very limited in what I can do, that's my biggest struggle and I want to learn some ways to improve my life.” | 8. Perception of severe physical functional limitations | (＋) Perceived threat of disease | Disease Perception and Personal Experience Building | Capacity |
|  |  | 9. Existence of a strong desire for improvement | (＋) Perceived threat of disease | Disease Perception and Personal Experience Building | Capacity |
| 6 | P4: “I have no difficulty using these apps, I use a variety of software daily.” | 10. Possession of daily software use experience | (＋) Prior technical experience | Disease Perception and Personal Experience Building | Capacity |
|  |  | 11. Perception of no difficulty in use | (＋) Prior technical experience | Disease Perception and Personal Experience Building | Capacity |
| 7 | P19: “I am quite good at operating my phone, I already have several software, more than enough.” | 12. Self-rated proficiency in technical operation | (＋) Prior technical experience | Disease Perception and Personal Experience Building | Capacity |
|  |  | 13. Existing software meets needs | (＋) Prior technical experience | Disease Perception and Personal Experience Building | Capacity |
| 8 | P8: “I'm a poor reader, … and when I use the app the interface, it is very easy to accidentally exit, I've been taught by volunteers many times before, but I still don't know how to operate it.” | 14. Presence of reading difficulties | (－) Cognitive decline | Dual Challenges of Cognitive Function and Physical Limitations | Capacity |
|  |  | 15. Prone to erroneous interface interaction | (－) Cognitive decline | Dual Challenges of Cognitive Function and Physical Limitations | Capacity |
|  |  | 16. Failure to master operation despite repeated instruction | (－) Cognitive decline | Dual Challenges of Cognitive Function and Physical Limitations | Capacity |
| 9 | P21: “The brain is not very good, looking at the screen is blurred, memory is bad, easy to forget; the cell phone is used only to make and answer calls. WeChat is not used.” | 17. Self-perceived cognitive decline | (－) Cognitive decline | Dual Challenges of Cognitive Function and Physical Limitations | Capacity |
|  |  | 18. Experience of blurred vision | (－) Cognitive decline | Dual Challenges of Cognitive Function and Physical Limitations | Capacity |
|  |  | 19. Short-term memory difficulties | (－) Cognitive decline | Dual Challenges of Cognitive Function and Physical Limitations | Capacity |
|  |  | 20. Use of only basic phone functions | (－) Cognitive decline | Dual Challenges of Cognitive Function and Physical Limitations | Capacity |
|  |  | 21. Avoidance of complex apps (e.g., WeChat) | (－) Cognitive decline | Dual Challenges of Cognitive Function and Physical Limitations | Capacity |
| 10 | P6: “I'm not well at the moment, I come over here to the nursing home to get well, and I feel that using these will increase the burden of my illness.” | 22. Actively resting due to poor health | (－) Restrictions on physical functioning | Dual Challenges of Cognitive Function and Physical Limitations | Capacity |
|  |  | 23. Fear that technology use increases physical burden | (－) Restrictions on physical functioning | Dual Challenges of Cognitive Function and Physical Limitations | Capacity |
| 11 | P23: “I mainly look at the computer because I think the phone is too small for my eyes and it's hard to look at it, it's hard to look at it twice with my eyes.” | 24. Preference for larger-screen devices | (－) Restrictions on physical functioning | Dual Challenges of Cognitive Function and Physical Limitations | Capacity |
|  |  | 25. Phone screen causes visual fatigue and discomfort | (－) Restrictions on physical functioning | Dual Challenges of Cognitive Function and Physical Limitations | Capacity |
| 12 | P14: “It's easy and I'd like to have more access to it.” | 26. Perception of technological convenience | (＋) Perceived ease of use | Digital Technology Integration and Life Adaptation | Capacity |
|  |  | 27. Expression of further use intention | (＋) Perceived ease of use | Digital Technology Integration and Life Adaptation | Capacity |
| 13 | P9: “I used to forget to take my medication all the time, but now I have reminders to know when I should take my medication.” | 28. History of forgetting medication due to memory lapses | (－) Cognitive decline | Dual Challenges of Cognitive Function and Physical Limitations | Capacity |
|  |  | 29. Technology reminder function effectively solves the problem | (＋) Integration of technology into daily life | Digital Technology Integration and Life Adaptation | Capacity |
| 14 | P2: “I think it's better for older people to read books than to look at their cell phones, there is gold in books.” | 30. Belief that traditional reading is superior to digital media | (－) Preference for traditional information resources | Digital Technology Integration and Life Adaptation | Capacity |
| 15 | P6: “I like to read newspapers, I've been reading them for more than 40 years, I'm used to it, I seldom have a cell phone.” | 31. Long-standing habit of reading newspapers | (－) Preference for traditional information resources | Digital Technology Integration and Life Adaptation | Capacity |
|  |  | 32. Infrequent phone use due to habit | (－) Preference for traditional information resources | Digital Technology Integration and Life Adaptation | Capacity |
| 16 | P6: “My wife tried it and said it was good and kept encouraging me to try it too.” | 33. Family recommendation after personal trial | (＋) Family support and encouragement | Access to and Utilization of Social Impact and Support Resources | Opportunities |
|  |  | 34. Receipt of persistent encouragement from family | (＋) Family support and encouragement | Access to and Utilization of Social Impact and Support Resources | Opportunities |
| 17 | P11: “My daughter told me last time that it was easy and would help me.” | 35. Family member indicated ease of operation | (＋) Family support and encouragement | Access to and Utilization of Social Impact and Support Resources | Opportunities |
|  |  | 36. Promise of assistance | (＋) Family support and encouragement | Access to and Utilization of Social Impact and Support Resources | Opportunities |
| 18 | P5: “Lao Zhuang shares his experience of using the app every day and encourages us to let us all try it, we all have to learn from him.” | 37. Peer daily experience sharing | (＋) Peer support | Access to and Utilization of Social Impact and Support Resources | Opportunities |
|  |  | 38. Peer active encouragement | (＋) Peer support | Access to and Utilization of Social Impact and Support Resources | Opportunities |
|  |  | 39. Peer behavior creates a modeling effect | (＋) Peer support | Access to and Utilization of Social Impact and Support Resources | Opportunities |
| 19 | P15: “They all think it's helpful to me and I want to try it too.” | 40. Perception of peer recognition of its utility | (＋) Peer support | Access to and Utilization of Social Impact and Support Resources | Opportunities |
|  |  | 41. Generation of trial intention influenced by peers | (＋) Peer support | Access to and Utilization of Social Impact and Support Resources | Opportunities |
| 20 | P8: “My kids won't let me use it, they always tell me to leave it alone, that it's too much trouble.” | 42. Family prohibition due to "trouble" concerns | (－) Overprotection by family members | Access to and Utilization of Social Impact and Support Resources | Opportunities |
| 21 | P15: “They say the techniques are too complicated and I'm sure I can't use them, but how do I know if I can use them if I haven't even tried them yet?” | 43. Family's negative capability prediction based on stereotype | (－) Overprotection by family members | Access to and Utilization of Social Impact and Support Resources | Opportunities |
|  |  | 44. Family prediction hinders trial opportunity | (－) Overprotection by family members | Access to and Utilization of Social Impact and Support Resources | Opportunities |
|  |  | 45. Feeling helpless and questioning family's prediction | (－) Overprotection by family members | Access to and Utilization of Social Impact and Support Resources | Opportunities |
| 22 | P12: “Depression is an illness that a lot of people don't understand, especially within nursing homes, they talk about you behind your back all the time.” | 46. Perception of widespread social misunderstanding of depression | (－) Stigmatization of mental illnesses | Access to and Utilization of Social Impact and Support Resources | Opportunities |
|  |  | 47. Experience of stigma (being talked about) in living environment | (－) Stigmatization of mental illnesses | Access to and Utilization of Social Impact and Support Resources | Opportunities |
| 23 | P2: “I don't have a smartphone, and these apps don't work at all.” | 48. Lack of necessary smart device | (－) Lack of digital equipment | Resource and Environmental Constraints and Facilitation | Opportunities |
| 24 | P24: “Mainly because of the lack of traffic, my cell phone is used take and make calls, and I don't use the other features until I have WIFI at home.” | 49. Mobile data cost as a constraint | (－) Lack of stable internet connection | Resource and Environmental Constraints and Facilitation | Opportunities |
|  |  | 50. Reliance on WiFi for advanced features | (－) Lack of stable internet connection | Resource and Environmental Constraints and Facilitation | Opportunities |
| 25 | P7: “It's always the same content and I don't want to use it anymore.” | 51. Perception of repetitive, non-updated app content | (－) Lack of personalization of content | Resource and Environmental Constraints and Facilitation | Opportunities |
| 26 | P15: “There are a lot of features, but I always feel like something is missing and the advice given to me is not tailored to my individual situation, it would be great if it could be adapted to my needs.” | 52. Perception of numerous but not thoughtful features | (－) Lack of personalization of content | Resource and Environmental Constraints and Facilitation | Opportunities |
|  |  | 53. Lack of individualized, tailored advice | (－) Lack of personalization of content | Resource and Environmental Constraints and Facilitation | Opportunities |
| 27 | P4: “I have found that seeing a doctor through my phone and not having to run to the hospital and stand in line is a real time and money saver.” | 54. Recognition of time-saving benefit of mHealth | (＋) Accessibility and convenience of medical resources | Resource and Environmental Constraints and Facilitation | Opportunities |
|  |  | 55. Recognition of cost-saving benefit of mHealth | (＋) Accessibility and convenience of medical resources | Resource and Environmental Constraints and Facilitation | Opportunities |
| 28 | P14: “Now that seeing a doctor and making an appointment with a doctor works on my phone, I don't need to go to the hospital machine to make an appointment, otherwise I would have to go there very early in the morning every time.” | 56. Recognition of appointment convenience via mHealth | (＋) Accessibility and convenience of medical resources | Resource and Environmental Constraints and Facilitation | Opportunities |
|  |  | 57. Avoidance of travel-related hassles and early morning queuing associated with traditional methods | (＋) Accessibility and convenience of medical resources | Resource and Environmental Constraints and Facilitation | Opportunities |
| 29 | P2: “I'm so old, I haven't even figured out how to use an old person's phone, how can I possibly use a smartphone.” | 58. Self-limitation due to advanced age | (－) Low self-efficacy | The Intertwined Influence of Beliefs, Emotions and Motivation | Motivation |
|  |  | 59. Reduced confidence due to past tech learning failures | (－) Low self-efficacy | The Intertwined Influence of Beliefs, Emotions and Motivation | Motivation |
| 30 | P20: “I'm afraid I don't have the confidence to finish this you said, without touching it, is it okay to let my son operate it.” | 60. Lack of confidence in completing the task | (－) Low self-efficacy | The Intertwined Influence of Beliefs, Emotions and Motivation | Motivation |
|  |  | 61. Tendency to depend on children as proxies | (－) Low self-efficacy | The Intertwined Influence of Beliefs, Emotions and Motivation | Motivation |
| 31 | P7: “I don't dare to use it, there are too many scammers online, in case I get all my money.” | 62. Intense fear of online scams | (－) Stigmatization of eHealth services | The Intertwined Influence of Beliefs, Emotions and Motivation | Motivation |
| 32 | P22: “Worried about information leakage, have to pay attention to the privacy aspect.” | 63. Concerns about personal information security and privacy leaks | (－) Stigmatization of eHealth services | The Intertwined Influence of Beliefs, Emotions and Motivation | Motivation |
| 33 | P13: “Even the app is complimenting me on a job well done, which gives me confidence to keep using it.” | 64. App's positive feedback (praise) brings sense of accomplishment | (＋) Positive feedback mechanisms | The Intertwined Influence of Beliefs, Emotions and Motivation | Motivation |
|  |  | 65. Positive feedback enhances confidence and continuity of use | (＋) Positive feedback mechanisms | The Intertwined Influence of Beliefs, Emotions and Motivation | Motivation |
| 34 | P13: “I can now complete it myself after the volunteer taught me how to operate it very carefully last time. This one should be designed to fit our older people, actually I don't like the color, too colorful, it's hard to look at it.” | 66. External guidance (volunteer) key to learning operation | (＋) Training and mentoring | The Intertwined Influence of Beliefs, Emotions and Motivation | Motivation |
|  |  | 67. Gained independent operation ability after mastery | (＋) Training and mentoring | The Intertwined Influence of Beliefs, Emotions and Motivation | Motivation |
|  |  | 68. Belief interface design (color) unsuitable for elderly aesthetics/vision | (＋) Aging-friendly design | The Intertwined Influence of Beliefs, Emotions and Motivation | Motivation |
| 35 | P16: “Provide training, guide us how to use it a bit, how to deal with the difficulties we encounter with cell phone things. For example, I don't want this advertisement to appear, to delete it, messy, sometimes I can't do it, so I go down and ask the young people to click it off.” | 69. Need for systematic usage training | (＋) Training and mentoring | The Intertwined Influence of Beliefs, Emotions and Motivation | Motivation |
|  |  | 70. Need for specific problem-solving guidance (e.g., ads) | (＋) Provide clear operating instructions | The Intertwined Influence of Beliefs, Emotions and Motivation | Motivation |
|  |  | 71. Reliance on external help (young people) for unsolvable problems | (＋) Training and mentoring / (＋) Provide clear operating instructions | The Intertwined Influence of Beliefs, Emotions and Motivation | Motivation |
| 36 | P3: “When I am in a bad mood, I always feel that there is no point in doing anything.” | 72. Depressive mood (sense of meaninglessness) directly weakens behavioral motivation | (－) Mood disorders | The Intertwined Influence of Beliefs, Emotions and Motivation | Motivation |
